# The Relationship between Maternal Ideation and Exclusive Breastfeeding Practice among Saudi Nursing Mothers: A Cross-Sectional Study

**DOI:** 10.3390/nu15071719

**Published:** 2023-03-31

**Authors:** Wafaa T. Elgzar, DaifAllah D. Al-Thubaity, Mohammed A. Alshahrani, Rasha M. Essa, Heba A. Ibrahim

**Affiliations:** 1 Department of Maternity and Childhood Nursing, Nursing College, Najran University, Najran 66441, Saudi Arabia; wtelgzar@nu.edu.sa (W.T.E.);; 2 Department of Clinical Laboratory Sciences, Applied Medical Sciences College, Najran University, Najran 66441, Saudi Arabia; 3 Department of Obstetrics and Gynecologic Nursing, Nursing College, Damanhour University, Damanhour 22514, Egypt

**Keywords:** exclusive breastfeeding, maternal ideation, knowledge, beliefs, self-efficacy, Saudia Arabia

## Abstract

All mortality risk factors are higher in non-breastfed infants compared to infants under five months of age who receive Exclusive Breastfeeding (EBF). Examining the predicting role of maternal ideation in EBF practices can help to direct and strengthen the cooperation between multidisciplinary healthcare providers to formulate multidisciplinary breastfeeding enhancement strategies. Methods: This correlational cross-sectional study investigates the relationship between maternal ideation and EBF practice among Saudi nursing mothers at Maternal and Children’s Hospital (MCH) in Najran, Saudi Arabia. The study incorporated 403 Saudi nursing mothers aged 6–12 months with healthy infants. The data collected using a questionnaire comprises demographic characteristics and obstetric history, the EBF Practice scale, and a maternal ideation scale. The data was collected from the beginning of November 2022 to the end of January 2023 and analyzed using I.B.M. version 22. Results: Breastfeeding initiation within one hour occurred among 85.1% of women, while 39.2% fed their newborn only colostrum during the first three days. EBF until six months was practiced by 40.9% of the participants day and night and on-demand (38.7%). Furthermore, 60.8% of the study participants had satisfactory overall EBF practices. The cognitive part of maternal ideation shows that 68.2% of the participants had adequate knowledge and 63.5% had positive beliefs regarding EBF practice. The maternal psychological ideation dimensions show that 81.4% had high EBF self-efficacy. The maternal social ideation dimensions showed that high injunctive and descriptive norms were present among 40.9% and 37.5%, respectively. In addition, healthcare providers (39.2%) had the most significant social influence, followed by husbands (30.5%). Binary logistic regression shows that the mother’s age, occupation, and education are the significant demographic predictors of satisfactory EBF practices (*p* < 0.05). All maternal ideation constructs were positive predictors of satisfactory EBF practices (*p* < 0.05). Conclusion: Maternal ideation constructs are positive predictors of satisfactory EBF practice and can be used to predict high-risk groups and plan for further intervention.

## 1. Introduction

The fourth-millennium development goal is concerned with decreasing child mortality. The World Health Organization (WHO) reported that 40% of under-five mortality occurred in under one month, and most occurred in the first week of age [1]. Sankar et al. conducted a systemic review to explore the relationship between satisfactory breastfeeding practice and infant and child mortality. They found that all mortality risk factors were higher in non-breastfed infants than in infants exclusively breastfed under five months of age. Furthermore, the risk for infection-related mortality is twofold higher in the non-breastfed infant than in the exclusively breastfed [2]. Breastfeeding has unquestionable benefits for infants and mothers, whether rich or poor. The infant breastfeeding-related benefits are numerous and long-lasting, such as decreasing infectious disease risk, a decreased risk for type 2 diabetes mellitus and obesity, a decreased risk for respiratory asthma, and increased child intelligence and adulthood intelligence later on [3,4,5]. In other words, breastfeeding is a child’s first inoculation against death, disease, and poverty and the most important and permanent investment in human physical, cognitive, and social capability [6]. Maternal benefits of breastfeeding include decreased breast and ovarian cancer risk, increased birth spacing, and decreased risk for type 2 diabetes and obesity.

Spotting on the breastfeeding benefits, which recommended only breastfeeding as the sole nutrient for the infant in the first six months of life with the allowance of prescribed medications, minerals, vitamins, and oral rehydration therapy. Complementary foods are introduced after six months, besides breastfeeding until two years of age [7]. The Saudi Ministry of Health issued policies to enhance the early initiation and continuation of breastfeeding exclusively until six months and complementarily until two years [8]. Saudi Arabia is a Muslim country; therefore, the Quran and Hadith are the main sources of legalization. In the Holy Quran, breastfeeding is a granted right for the infant until two years of age [9]. Although the Saudi Ministry of Health paid great attention to the early initiation and continuation of EBF, the rate is still lower than expected. In Saudi Arabia, the EBF rate varied by region, ranging from 0.8% to 43.9%; in a systemic review involving 17 studies [10] worldwide, the WHO stated that less than 50% of infants have EBF [7].

The Saudi Ministry of Health paid great attention to increasing public awareness of breastfeeding, but the rate is still low. This may highlight a question about the effectiveness of such educational interventions. The educational interventions have to be built on strong subject-related factors and address the target group’s needs. Therefore, the most important step in a successful educational program is determining the target group’s needs and the predictors of satisfactory breastfeeding practice. Factors associated with EBF practices are multiple and have a complicated nature. Some factors are not modifiable, such as demographic and obstetrical variables, and many others are modifiable. Modifiable factors are breastfeeding knowledge, beliefs, self-efficacy, the common norms and patterns of EBF in the community, social norms, and significant others who can influence the women’s behaviors [11]. Modifiable factors are the milestones that can help educational programs produce behavior changes and stick to satisfactory EBF practices.

The maternal ideation model is one of the most important models that can be used to predict satisfactory EBF practice. Specifically, women with low maternal ideation, inadequate knowledge, negative beliefs, low self-efficacy, and low injunctive and descriptive norms are at a higher risk of unsatisfactory outcomes. The EBF ideation model tried to explain how new behaviors or practices can spread throughout the community through social interactions and communication among individuals and groups [12]. The ideation model of strategic communication and behavior change primarily focuses on behavioral and social changes toward positive health behaviors [13]. Interventions for improving breastfeeding based on the ideation model should focus on predicting the cognitive, psychological, and social dimensions of breastfeeding. Cognitive aspects of behavior change include women’s knowledge and beliefs regarding breastfeeding. Psychological aspects include breastfeeding self-efficacy, or how the mother evaluates her ability to practice EBF effectively. Lastly, the social dimension of the ideation models includes injunctive norms, descriptive norms, and social influences. The injunctive norms evaluated the woman’s trust in the benefits of breastfeeding for herself and her infant. Descriptive norms evaluated the women’s perceptions of community norms regarding breastfeeding. The social influence evaluation evaluated the significant person or social network who may influence the women’s breastfeeding practices. These people may be the husband, mother-in-law, friends, and health care provider. The ideation model is based on the assumption that a person with more enabling ideation factors can effectively manage behavior change to a more positive one [11].

The roles of ideation factors in predicting behavior change were examined for cancer care [13], contraceptive use [14], suicide [15], and malaria care [16], but rarely for breastfeeding in Nigeria [11]. Examining the predicting role of maternal ideation in EBF practices can help direct and strengthen the cooperation between multidisciplinary healthcare providers in formulating multidisciplinary breastfeeding enhancement strategies. Breastfeeding enhancement strategies can be implemented with the cooperation of breastfeeding specialists, pediatricians, psychologists, nurses, and social workers. The maternal ideation cognitive part (knowledge and beliefs) can be enhanced through educational programs provided by breastfeeding specialists, pediatricians, and nurses. Furthermore, the psychological maternal ideation dimension (breastfeeding self-efficacy) can be improved through educational and psychological interventions provided by psychologists. Lastly, the social part of the maternal ideation model (social influence, injunctive, and descriptive norms) can be enhanced through a social and educational intervention provided by social workers in cooperation with breastfeeding specialists, pediatricians, and nurses, all using different media. It can also help with EBF enhancement at national and subnational levels by accessing the whole community through all dimensions of the ideation model of strategic communication and behavioral change.

## 2. Materials and Methods

### 2.1. Study Design

This correlational cross-sectional study investigated the relationship between maternal ideation and EBF practice among Saudi nursing mothers in the Maternal and Children’s Hospital (MCH).

The operational definitions are: EBF gives the infant breast milk only for the first six months of life, in addition to prescribed medications, minerals, oral rehydration therapy, and vitamins [17].

Unsatisfactory EBF practice is below-average (≤10) on the EBF practice scale, and satisfactory EBF practice is above-average (>10).

Maternal ideation is the complex and unique decision-making process that leads to behavioral changes, and it incorporates cognitive (knowledge and beliefs), psychological (self-efficacy), and social (injunctive norms, descriptive norms, and social influence) dimensions related to EBF [11].

### 2.2. Study Setting

The study was conducted in the outpatient department of the MCH Hospital in Najran, Saudi Arabia. The included clinics are immunization, pediatrics, and breastfeeding counseling. MCH is the only hospital in Najran City that provides maternal and child health care; therefore, it serves a large population. Najran city is the capital of the Najran region in southwestern Saudi Arabia. Najran City has many cultural and traditional beliefs about the maternity cycle and breastfeeding [18].

### 2.3. Study Participants

The study incorporated 403 Saudi nursing mothers with infants free from breastfeeding contraindications and aged 6–12 months; those aged 18 years and older can read, write, and accept participation in the study.

#### 2.3.1. Sample Size and Sampling Procedures

The sample size was calculated using the Cohran formula [19]. The EBF prevalence in Saudi Arabia varies by region; it ranges from 0.8 to 43.9%, according to a systemic review by Al Junaid et al. [10]. Therefore, the EBF rate used in the sample size calculation is 43.9%. The formula used in the calculation is:

$$n=\frac{Z^{2}Pq}{e^{2}}$$

where *n* is the required sample size, *Z* is the normal standard deviation at a 95% confidence level (1.96), and *P* is the estimated proportion of the target population, estimated to be 43.9%; according to Al Junaid et al., the sample size is 378 after adding 15% to compensate for the anticipated sample loss of *n* = 435.

#### 2.3.2. Sampling Technique and Procedures

**Figure 1:**
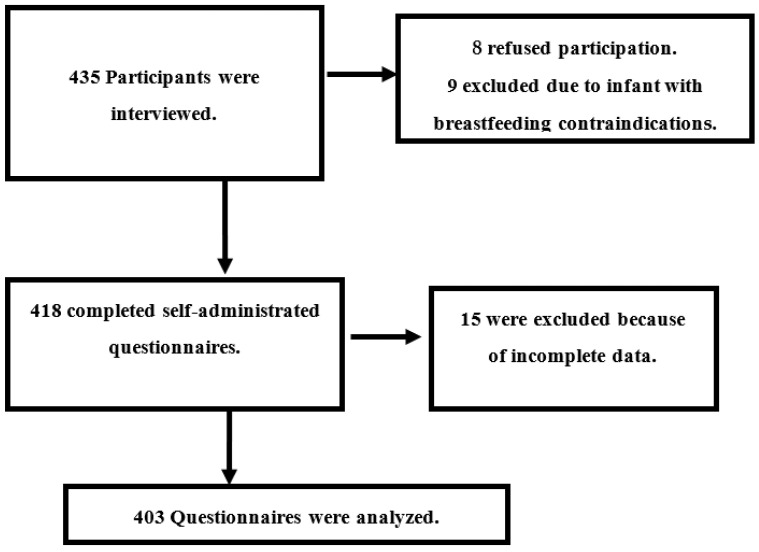
Participants’ flow chart.

A systemic random sampling was utilized to recruit the participants. The suitable clinics for data collection were three pediatric clinics, an immunization clinic, and a breastfeeding counseling clinic. According to the clinic registry system, there are a total of 5 clinics with an average follow-up rate of 75 cases (15 for each clinic) per day in the morning and afternoon shifts. The data collection team was composed of three researchers; each researcher determined that he could collect a minimum of five cases daily; the total is 15 cases per day for the whole team, three days per week. The sampling interval was determined by dividing the follow-up rate by the expected total recruited cases daily, and it was 75/5 = 5. The starting point was picked randomly from a ball containing papers numbered 1–5, then an interval of 5 was applied. The participants were picked from the waiting area for the five clinics, which ran three days per week for 12 weeks from November 2022 to January 2023. If the selected participant refused participation or was illegible, the next case was included, and the sample interval was maintained. The study participants were distributed according to the following chart (Figure 1).

### 2.4. Study Measurement Tools 

The study measurement tools are organized in a self-reported questionnaire that was developed after an intensive literature review. The questionnaire comprises three parts: demographic characteristics and obstetric history; EBF practice scale; and maternal ideation scale.

Part I consists of demographic characteristics and obstetric histories. It collects the mothers’ age, occupation, education, and husband’s education, as well as their monthly income. Obstetric history includes parity, mode of delivery, complications of the last delivery, duration of pregnancy for the last child, and the number of living children.

Part II is the self-reported EBF practice scale. It was developed by researchers and comprises ten statements that evaluated the initiation and different aspects of breastfeeding practices, guided by previous studies [17,20]. The scale items were dichotomous (yes or no) questions, where yes scored “one” and no scored “zero”, The total scale score was 10, and the participant was considered to have unsatisfactory (0–5) or satisfactory (6–10) practice based on her score. Cronbach’s alpha coefficient for the practice scale was 0.87, indicating high reliability.

Part III is the maternal ideation evaluation scale. Maternal ideation is composed of three main categories, which are cognitive (knowledge and beliefs), psychological (self-efficacy), and social (injunctive norms, descriptive norms, and social influence) [11].

The maternal breastfeeding knowledge was assessed using the Gender-Friendly Breastfeeding Knowledge Scale (GFBKS). It was validated by Gupta et al. and found to be highly reliable (r = 0.787). The scale incorporated 18 items rated on a 5-point Likert scale as false = 1, maybe false = 2, do not know = 3, may be true = 4, and true = 5. The total score for the 18 items ranged from 18 to 90. Based on her score, the mother was classified as having inadequate (18–54) or adequate knowledge (55–90) [21].

Mothers’ beliefs regarding breastfeeding are adapted from the beliefs about breastfeeding questionnaire. The questionnaire consisted of 8 items rated on a 5-point Likert scale ranging from strongly disagreeing (1 point) to strongly agreeing (5 points). The total questionnaire score ranged from 8 to 40; the negative belief score ranged from 8 to 24; and the positive belief ranged from 25 to 40. The questionnaire indicated good internal consistency, which ranged from (r = 0.73–0.77) [22].

The psychological part of maternal ideation, mainly self-efficacy, was evaluated using the Breastfeeding Self-Efficacy Scale—Short Form (BSES-SF). Dennis created it in 2003 to evaluate postpartum women’s breastfeeding confidence. The BSES-SF was rated on a 5-point Likert scale ranging from 1 (not at all confident) to 5 (always confident). The total score ranged from 14–70; low self-efficacy is considered from 14–42 and high self-efficacy from 43–70 [23]. According to Amini et al., the BSES-SF internal consistency was (r = 0.910) [24].

The injunctive norms evaluated the mother’s trust in breastfeeding benefits for herself and her infant. The injunctive norms were evaluated using four items rated on a five-point Likert scale, resulting in a total injunctive norms score (4–20). The mother was considered to have low (4–12) or high (13–20) injunctive norms according to her total score for the four items [11].

Descriptive norms evaluated the mother’s perception of the community norms regarding breastfeeding. It was evaluated through three items rated on a 5-point Likert scale ranging from strongly disagreeing (1 point) to strongly agreeing (5 points). The total descriptive norms score ranged from 3–15, and the woman was considered to have low (3–9) or high (10–15) descriptive norms according to her total score for the three items [11].

The social influence is evaluated using one multiple-choice question to evaluate who, besides the mother, can affect her decision regarding breastfeeding. The answers were no one, husband, mother-in-law, and health care provider [11].

### 2.5. Data Collection Procedures and Techniques

The data was collected from the beginning of November to the end of January 2023. At the beginning of the data collection interview, the researcher explained the study purpose, expected outcome, participants’ role, data confidentiality, and the right to refuse participation without any penalties or consequences on the care provided. If the mother accepted participation, the questionnaire was given to her after she signed the informed consent. The data collector was present during the data collection process to clarify concerns and answer the participants’ questions. After completing the questionnaire, the participant’s medical records were used to confirm the basic data.

### 2.6. Data Quality Control

The three data collectors were researchers with good experience in data collection, sampling techniques, and research ethics. Before data analysis, the questionnaires were carefully checked for missing data; 15 cases were excluded.

### 2.7. Ethical Approval

At first, the research proposal was approved by the deanship of scientific research at Najran University; then, another approval was obtained from the Najran Health Affairs Ethics Committee, an ethical approval (I.R.B. Log Number 2023-02 E). Approval to start data collection was also obtained from the MCH head. In addition, during data collection, informed consent was obtained from each participant after explaining the study’s purpose. Data confidentiality, anonymity, and the right to refuse participation without any penalties were emphasized to the participants.

### 2.8. Data Analysis 

Data analysis was performed using the Statistical I.B.M. software, version 23 (I.B.M. Corp., Armonk, NY, USA). The data were checked for normal distribution and missing data. Sheets containing missing data were omitted from the analysis. The participants’ basic data, obstetric history, and maternal ideation constructs were described using numbers, percentages, and mean ± SD. The total EBF knowledge, beliefs, self-efficacy, injunctive norms, and descriptive norms were obtained by summing items. The predictors of satisfactory EBF practice were examined using binary logistic regression. Among the independent variables, the participant’s age, total B.F. knowledge, total B.F. beliefs, self-efficacy, injunctive norms, descriptive norms, gravidity, and the number of living children are continuous. Furthermore, occupation, mother’s education, husband’s education, monthly income, gravidity, mode of delivery of the last child, social influence, and duration of pregnancy are all categorical variables. The first category was the reference for all categorical variables. All factors were tested for multicollinearity before the regression model. The final model was tested with the Cox and Snell R-Squared goodness of fit test. Statistically significant findings were considered at *p* ˂ 0.05.

## 3. Results

The participants’ demographic characteristics are illustrated in Table 1. More than three-quarters (82.1%) of the study participants were aged 20–35, with a mean age of 28.95. Furthermore, 62.5%, 66.3%, and 67.7% are working, university-educated mothers and married to highly educated husbands, respectively. In addition, more than half (52.4%) of the study participants had enough monthly income, but only 10.2% could save.

Obstetric history shows that 67.7% and 70.2% of the participants were multiparous and had a history of previous vaginal delivery, respectively. Furthermore, 83.1% and 90.6% of women suffered from complications during the last delivery and had full-term pregnancies, respectively. The mean number of living children for the study participants was 2.732 (Table 2).

Self-reported EBF practices are illustrated in Table 3. Breastfeeding initiation within one hour occurred among 85.1% of women, while 39.2% fed their newborns only colostrum during the first three days of their lives. EBF until six months of life was practiced by 40.9% of the participants. Breastfeeding was practiced by 38.7% of women at all hours of the day and night. Nearly two-thirds of the participants ensured that their infant was properly latched on for the whole feeding (65%), determined that the baby got enough milk (64%), and fed their infant from the two breasts interchangeably (63.5%). In addition, 54.6% can comfortably breastfeed in the presence of family members. Lastly, 67.2% and 65.3% of the study participants maintained comfortable positions and performed eructation after each feeding, respectively. Furthermore, 60.8% of the study participants had satisfactory EBF practices, and 39.2% had unsatisfactory practices.

The cognitive part of maternal ideation shows that 68.2% of the participants had adequate knowledge and 63.5% had positive beliefs regarding breastfeeding practice. The maternal psychological ideation dimension shows that 81.4% had high breastfeeding self-efficacy. The maternal social ideation dimensions showed that high injunctive and descriptive norms were present among 40.9% and 37.5%, respectively. In addition, the most significant social influence is provided by health care providers (39.2%), followed by husbands (30.5%) (Table 4).

Binary logistic regression shows that the mother’s age, occupation, and education are all significant demographic predictors of EBF practices. An increase in the maternal age by one year increases the probability of satisfactory EBF practice by 1.2 times [AOR = 1.267 (1.109–1.449), *p* = 0.001]. Furthermore, being a housewife and university educated too much increased the probability for satisfactory EBF [AOR = 13.341 (2.249–39.249), *p* = 0.004] and [AOR = 3.218 (1.861–10.682), *p* = 0.008]. Obstetric history, parity, previous delivery mode, last pregnancy duration, and the number of living children were predictors of satisfactory EBF practices. Previous history of cesarean delivery [AOR = 0.239 (0.104–0.547), *p* = 0.001] and having preterm infants [AOR = 0.846 (0.780–0.898), *p* = 0.000] decreased the probability of satisfactory EBF practices, when taking vaginal delivery and a full-term infant as a reference. Moreover, being multiparous increased the probability of satisfactory EBF practices compared to primiparous mothers [AOR = 3.716 (1.214–16.290), *p* = 0.027]. Furthermore, an increase in the number of children by one increases the probability of satisfactory EBF practices by 4.3 times [AOR = 4.317 (1.806–8.236), *p* = 0.000]. (Table 5).

Binary logistic regression shows that all maternal ideation constructs were positive predictors of satisfactory EBF practices. For the cognitive diminution of maternal ideation, an increase in maternal knowledge [AOR = 5.96 (1.968–17.787), *p* = 0.002] and beliefs [AOR = 1.76 (1.016–1.821), *p* = 0.033] increases the mother’s probability to practice satisfactory EBF by 5.9 and 1.7 times, respectively. For the psychological dimension of maternal ideation, an increase in self-efficacy [AOR = 1.877 (1.201–1.939), *p* = 0.000] of one degree nearly doubled the probability of practicing EBF. For the social dimension, the husband’s [AOR = 1.612 (0.732–3.877), *p* = 0.038] and health care provider’s [AOR = 1.661 (1.066–4.016), *p* = 0.047] influence increased the mother’s probability to have satisfactory EBF practice when compared to having none. Lastly, a one-degree increase of injunctive [AOR = 4.132 (1.656–8.874), *p* = 0.000] and descriptive norms [AOR = 5.547 (2.018–10.815), *p* = 0.000] increased the mother’s probability of practicing satisfactory EBF by 4.13 and 5.54 times, respectively. Cox and Snell R-Square showed that the current model could predict 57.8% of the satisfactory EBF practices (Table 6).

## 4. Discussion

Breastfeeding in Saudi Arabia is influenced by a range of religious and cultural factors [9]. In addition, the benefits of breastfeeding are undeniable and supported by the Ministry of Health’s educational efforts. Therefore, the challenge is to achieve and maintain satisfactory EBF practices, not only initiation. Satisfactory EBF practices were found among approximately two-thirds of the current study participants, with a relatively high early initiation rate among a large proportion of the study participants. However, there is a discrepancy between a very high breastfeeding initiation rate within the first hour (85.1%) and exclusive breastfeeding being continued only in 40.9% of cases, despite a generally positive maternal approach to breastfeeding supported by Muslim religious beliefs. The discrepancy between the early initiation rate and continuation of EBF can be explained by two main factors. First, in MCH, numerous policies support early initiation of EBF within one hour; therefore, early initiation of breastfeeding is an important task for healthcare providers in the delivery room and must be documented in the patient record. The second factor that may negatively affect EBF continuation is related to some traditions related to breastfeeding, such as giving some herbal teas for baby colic. Significant people in the woman’s social network may advise her to give some herbal remedies or complementary bottle feeding during the first six months of life because they think that breast milk is insufficient [25]. Previous studies have reported the initiation rate to be high in different Saudi regions, but the continuation rate is still much lower than recommended [26,27,28]. Saudi women are guided by their religious beliefs to initiate breastfeeding but have challenges with its continuation. Most Saudi women discontinued breastfeeding against their wishes due to work and social barriers. It is worthy of note that in Saudi Arabia, the maternity leave period is limited to only 45–70 days (1.5–2.3 months) by law, which is too short for an employed mother to practice EBF for six months, which may force the mother to appoint babysitters and make their EBF continuation a challenge. The social challenge for lactating mothers who want to continue breastfeeding is how to handle social pressure and traditions related to EBF. Their close relatives mostly advise them to give herbs or bottle-feed the infant to promote better growth. Colostrum is fed to about two-fifths of the current study participants, and EBF is practiced day and night. Saudi women were found to have good knowledge about colostrum and reported a high preference to give only colostrum during the first three days of the newborn’s life [29,30]. However, they may be pushed by baby illness, maternal exhaustion, a lack of social support, and some traditions to give the baby herbal drinks or formula milk and decrease the amount of colostrum feeding or avoid feeding it at all. The women also reported giving bottle feeding with breastfeeding from three months of age, and they reported insufficient breast milk or returned work as the main factors for introducing bottle feeding [28,29]. Therefore, the factors associated with EBF practices are multiple and complicated, containing cognitive, psychological, and social dimensions, which are the main components of the maternal ideation model.

In the current study, maternal ideations play an important role in EBF practices, where all maternal ideation constructs are positive predictors of satisfactory EBF practices. Specifically for the cognitive domain, an increase in maternal knowledge and beliefs regarding breastfeeding by one grade increases the women’s probability to practice satisfactory EBF by 5.9 and 1.7 times, respectively. The maternal ideation role in breastfeeding was investigated by Anaba et al. in Nigeria. They reported on the important role of maternal ideation in predicting E.P.F. practice. They added that various cognitive, emotional, and social dimensions of maternal ideation significantly influenced the woman’s breastfeeding decisions. Maternal knowledge regarding breastfeeding benefits, appropriate timing, and their beliefs regarding colostrum benefits (cognitive), women’s breastfeeding self-efficacy (emotional), and perceived breastfeeding norms (social) were the most significant predictors of breastfeeding initiation in Nigeria [11].

Knowledge is well documented in previous national [31,32] and international studies [17,33,34] to be strongly linked to satisfactory EBF practices. The link between breastfeeding knowledge and satisfactory EBF practices seems logical because if the woman has to adopt any behavior, she should have sufficient knowledge to fit in and gain harmony with her previous mental schema. Therefore, many previous studies reported significant improvements in EBF practices after an educational program [17,35]. Most studies that evaluated breastfeeding knowledge also evaluated beliefs and may explore the link between breastfeeding knowledge and attitude [17,35]. This unique link presents the cognitive part of maternal ideation as an important component of breastfeeding practices. A previous study concluded that in an ambiguous situation that requires a decision, like EBF, there is a unique mediating role for knowledge in belief formulation, which consequently affects breastfeeding practice [36]. Saudi women’s beliefs regarding breastfeeding are strongly affected by religious instructions from the Holy Quran and Hadith. In the Holy Quran, the infant is granted the right to breastfeed for two years; therefore, Muslim women have positive beliefs regarding breastfeeding, even if there are barriers to it [9].

Psychological readiness for breastfeeding is an important pillar in achieving satisfactory EBF practices, which reflects the importance of the psychological dimension of the maternal ideation model [11]. Breastfeeding self-efficacy reflects the woman’s confidence in her ability to exclusively breastfeed her infant, and it acts as an important modifiable factor for satisfactory EBF practices. The current study showed that an increase of one grade in the woman’s breastfeeding self-efficacy score could nearly double the probability of EBF practices. In this regard, Brockway et al. conducted a systemic review and meta-analysis of eleven studies exploring breastfeeding self-efficacy’s role in EBF practices. They found that a one-point increase in the breastfeeding self-efficacy score can increase the probability of EBF by 10% compared with the control group [37]. In addition, breastfeeding self-efficacy was identified as a golden factor associated with the early initiation and continuation of EBF [38]. Psychological parts of the maternal ideation model (breastfeeding self-efficacy) represent the internal power that can help a woman use the available resources and support systems to enhance her EBF practices. Therefore, there is a connection between breastfeeding self-efficacy and the social part of the maternal ideation model.

The social part of the maternal ideation model incorporates social influence, injunctive norms, and descriptive norms. Social influencers represent the mother’s support network, and they, other than herself, influence her decision to practice EBF. In the current study, husbands and health care providers’ support increased the women’s probability of having satisfactory EBF practices when compared to having none. A previous interventional study explored the role of husband support in breastfeeding practice. They found that increasing the husband’s knowledge could improve the mother’s knowledge and attitude toward EBF. On the other side, a motivated husband can provide physical and psychological support to his wife and help her overcome breastfeeding challenges [39]. Therefore, improving couples’ knowledge and attitude can significantly improve satisfactory EBF practices. Another study elaborated that the husband’s role in breastfeeding is not limited to his knowledge and attitude toward breastfeeding but also extended to women’s empowerment to provide satisfactory EBF practices. As the husband represents the power source in Saudi families, his positive attitude and support of EBF practices can shape the mother’s subjective criteria related to breastfeeding [40]. Healthcare providers are the first influencers who can prepare the woman during pregnancy regarding breastfeeding and help the woman during the postpartum period to initiate it early. In addition, they are counselors, educators, and motivators for the woman for EBF. Healthcare providers can also tailor and apply breastfeeding-friendly policies [28]. The role of health care providers is not only limited to women’s education regarding breastfeeding; it also extends to the husband, family, and the whole community. Healthcare providers conducted numerous health education and counseling interventions to increase community awareness and social support for breastfeeding. McFadden et al. explored the role of healthcare providers’ support, education, and monitoring in EBF initiation and continuation. They concluded that education, counseling, and continuous home visits by trained healthcare providers during the antenatal and postnatal period significantly increased the percentage of satisfactory EBF practices [41].

Lastly, a one-degree increase in injunctive and descriptive norms increased women’s probability of satisfactory EBF practices by 4.13 and 5.54 times, respectively. The injunctive norms evaluated the woman’s trust in breastfeeding benefits for herself and her infant. Descriptive norms evaluated the women’s perceptions of community norms regarding breastfeeding. Being a Muslim generates a positive perception and tradition regarding breastfeeding anywhere and generates a descriptive norm that all Muslim women should breastfeed their infants. Kamoun and Spatz conducted a study to investigate the Islamic tradition’s influence on breastfeeding practice among African American Muslims in West Philadelphia compared to non-Muslim African Americans. They concluded that Muslim women have a higher perception of breastfeeding benefits for mothers and infants. Most Muslim women have a positive attitude and higher breastfeeding self-efficacy [42]. The present study results are also in line with Anaba et al., who reported a significant association between E.P.F. practices and injunctive norms [11].

Breastfeeding is common among Saudi women but not exclusive. Saudi tradition enforces the introduction of complementary feeding and traditional herbs for the infant. Therefore, most women feed their infants, as reflected in their injunctive and descriptive norms [43].

In conclusion, Cox and Snell R-Square showed that 57.8% of satisfactory EBF practices could be predicted by the maternal ideation model for behavior change. Therefore, this model needs to be considered while designing and implementing breastfeeding educational interventions. The model tackles the most important cognitive (knowledge and beliefs), psychological (breastfeeding self-efficacy), and social (social influences, injunctive norms, and descriptive norms) dimensions that may significantly enhance EBF practices. Anaba et al. recommended that the utilization of the maternal ideation model in breastfeeding programs can promote more sustainability of positive breastfeeding practices [11].

### Strengths and Limitations

This study discussed an important behavior change model that is rarely studied concerning breastfeeding. The sample size was estimated based on a 95% confidence interval, and a systemic random sampling procedure was used to engage the participants, which improved the results’ generalizability. The AOR was estimated to perfectly assess the role of each predictor in satisfactory EBF practice and the role of each maternal ideation construct. Yet, this research has some limitations. The data gathered in this study is self-reported, which raises the potential for recall bias or the desire for social acceptance. In addition, replication of the current study is important to compare the differences between the different Saudi regions.

## 5. Conclusions

Around two-thirds of the study participants had satisfactory EBF practices with a relatively high early initiation rate. The mother’s age, occupation, and education are significant demographic variables for satisfactory EBF practices. Moreover, parity, previous delivery mode, duration of the last pregnancy, and the number of living children were predictors of satisfactory EBF practices. Furthermore, knowledge and beliefs under cognitive maternal ideation, self-efficacy under psychological maternal ideation, injunctive and descriptive norms, and social influence under social maternal ideation were positive predictors of satisfactory EBF practices. The maternal ideation model could provide important insight to healthcare providers, policymakers, and health program designers about important modifiable and satisfactory EBF predictors.

## Figures and Tables

**Table 1:** Participants’ sociodemographic data (*n* = 403).

Sociodemographic Data	*n* (403)	%
**Age** (years)		
– ˂20 year	14	3.5
– 20–35	331	82.1
– ≥36	58	14.4
**Mean ± SD**	28.95 ± 7.64	
**Occupational status**		
– Housewife	151	37.5
– Working	252	62.5
**Woman’s education**		
– Read and write	20	5.0
– Secondary school	116	28.8
– University or postgraduate	267	66.3
**Husband’s education**		
– Read and write	10	2.5
– Secondary school	120	29.8
– University or postgraduate	273	67.7
**Monthly income**		
– Not enough	151	37.5
– Enough	211	52.4
– Enough and can save	41	10.2

**Table 2:** Participants’ obstetric history and breastfeeding practice (*n* = 403).

Participants’ Obstetric History and Breastfeeding Practices	*n*	%
**Parity**		
- Primiparous	130	32.3
- Multiparous	273	67.7
**Mode of delivery**		
- Vaginal delivery	283	70.2
- Cesarean section	120	29.8
**Complications of last delivery**		
- No	335	83.1
- Yes	68	16.9
**Duration of pregnancy with the last child**		
- Full-term	365	90.6
- Preterm	38	9.4
**Number of living children (mean ± SD)**	2.732 ± 2.024	

**Table 3:** Self-reported EBF practice (*n* = 403).

	Practice Items	Yes		No	
		N	%	N	%
1	I started to breastfeed my infant during the first hour of postpartum.	343	85.1	60	14.9
2	I fed my infant only colostrum during the first three days.	158	39.2	245	60.8
3	I feed my infant only breast milk until 6 months of age.	165	40.9	238	59.1
4	I feed my infant day and night and on demand.	156	38.7	247	61.3
5	During feeding, I ensured that my infant was properly latched on for the whole feeding.	262	65.0	141	35.0
6	During feeding, I determined that my baby got enough milk.	258	64.0	145	36.0
7	I feed my infant from both breasts interchangeably.	256	63.5	147	36.5
8	I comfortably breastfeed with my family members present.	220	54.6	183	45.4
9	I maintained comfortable positions for my infant and me during feeding.	271	67.2	132	32.8
10	I performed eructation for my infant after each feeding.	263	65.3	140	34.7
		Unsatisfactory		Satisfactory	
	Total EBF practice.	158	39.2	245	60.8

**Table 4:** Participants’ breastfeeding maternal ideation (*n* = 403).

Ideational Dimension	Domain	*n*	%
**Cognitive**	**Knowledge**		
	Inadequate (18–54)	128	31.8
	Adequate (55–90)	275	68.2
	**Beliefs**		
	Negative (8–24)	147	36.5
	Positive (25–40)	256	63.5
**Psychological**	**Self-efficacy**		
	Low (14–42)	75	18.6
	High (43–70)	328	81.4
**Social**	**Injunctive norms**		
	Low	238	59.1
	High	165	40.9
	**Descriptive norms**		
	Low	252	62.5
	High	151	37.5
	**Social influence**		
	No one	69	17.1
	Husband	123	30.5
	Mother-in-law	53	13.2
	Health care provider	158	39.2

**Table 5:** Binary logistic regression analysis of the demographic and obstetric history predictors of satisfactory EBF practice (*n* = 403).

Predictors	EBF Practice	
	AOR (95% CI)	*p*
**Occupational status**		
– Working	Ref	
– Housewife	13.341 (2.249–39.249)	0.004 *
**Education**		0.028
– Read and write	Ref	
– Secondary school	1.037 (0.964–1.116)	0.324
– University or postgraduate	3.218 (1.861–10.682)	0.008 *
**Husband’s education**		0.621
– Read and write	Ref	
– Secondary school	0.958 (0.954–1.046)	0.652
– University or postgraduate	0.942 (0.964–1.133)	0.715
**Monthly income**		0.814
– Enough and can save	Ref	
– Enough	0.928 (0.363–2.372)	0.775
– Not enough	1.114 (0.393–3.163)	0.839
**Parity**		
- **Primiprous**	Ref	
- **Multiparous**	3.716 (1.214–16.290)	0.027 *
**Mode of delivery**		
- Vaginal delivery	Ref	
- Cesarean section	0.239 (0.104–0.547)	0.001 *
**Complications during last delivery**		
- Yes	Ref	
- No	1.169 (0.484–2.823)	0.729
**Duration of pregnancy**		
- Full-term	Ref	
- Preterm	0.846 (0.780–0.898)	0.000 **
**Age**	1.267 (1.109–1.449)	0.001 *
**Number of living children (mean ± SD)**	4.317 (1.806–8.236)	0.000 **

AOR: Adjusted Odd Ratio. CI: Confidence Interval. * significant at *p* ˂ 0.05. ** significant at *p* ˂ 0.001.

**Table 6:** Binary logistic regression analysis for the predictive relation of the maternal ideation model on the satisfactory EBF practice (*n* = 403).

	Predictors	EBF Practice	
		AOR (95% CI)	*p*
**Cognitive**	- Knowledge	5.916 (1.968–17.787)	0.002 *
	- Beliefs	1.76 (1.016–1.821)	0.033 *
**Psychological**	**Self-efficacy**	1.877 (1.201–1.939)	0.000 **
**Social**	**Social influence**		0.016 *
	- No one	Ref	
	- Husband	1.612 (0.732–3.877)	0.038 *
	- Mother-in-law	0.581 (0.268–1.268)	0.171
	- Health care provider	1.661 (1.066–4.016)	0.047 *
	**Injunctive norms**	4.132 (1.656–8.874)	0.000 **
	**Descriptive norms**	5.547 (2.018–10.815)	0.000 **
	−2 Log likelihood (357.778)	Cox and Snell R-Square (0.578)	Nagelkerke R-Square (0.802)

AOR: Adjusted Odd Ratio. CI: Confidence Interval. * significant at *p* ˂ 0.05. ** significant at *p* ˂ 0.001.

## Data Availability

The data will be made available by the corresponding author upon reasonable request.

## Abbreviations

AOR	Adjusted Odd Ratio
EBF	Exclusive Breastfeeding
GFBKS	Gender-Friendly Breastfeeding Knowledge Scale
MCH	Maternal and Children’s Hospital
